# Perceptions and attitudes of healthcare workers towards the use of digital facial recognition application in a health setting in Uganda: An exploratory pilot study

**DOI:** 10.1371/journal.pone.0337691

**Published:** 2025-11-25

**Authors:** Patrick Kaggwa, Juliet Nabbuye Sekandi, Mcdonald Kerone Adenike, Peter Nabende, Sarah Nabukeera, Kenneth Kidonge Katende, Esther Buregyeya, Nazarius Mbona Tumwesigye

**Affiliations:** 1 Department of Epidemiology and Biostatistics, School of Public Health, College of Health Sciences, Makerere University, Kampala, Uganda; 2 Global Health Institute, College of Public Health, University of Georgia, Athens, Georgia, United States of America; 3 Department of Research and Data, Joint Clinical Research Centre, Kampala, Uganda; 4 Department of Disease Control and Environmental Health, School of Public Health, College of Health Sciences, Makerere University, Kampala, Uganda; 5 Department of Information Systems, School of Computing and Informatics Technology, College of Computing and Information Science, Makerere University, Kampala, Uganda; University of Science and Technology, YEMEN

## Abstract

**Background:**

Unique patient identification is often challenging in healthcare systems, especially in low- and middle-income countries. Digital facial recognition is a promising alternative to traditional identification methods. This pilot study explores the perceptions and attitudes of healthcare workers towards using facial recognition technology in a healthcare setting in Uganda.

**Methods:**

We conducted an explorative qualitative study using key informant interviews with healthcare workers in Kampala, Uganda, to assess perceptions and attitudes towards digital facial recognition. We interviewed a total of 10 healthcare workers, including five doctors and five nurses, aged 20–39 years, with at least one year of professional experience. A trained interviewer provided a brief overview and demonstration of the facial recognition application and then used an open-ended interview guide to elicit responses about perceptions and attitudes. The interviews were audio recorded and transcribed verbatim. Data obtained from Key Informant Interviews were manually analyzed using thematic content analysis.

**Results:**

Overall, the healthcare workers perceived digital facial recognition as a more effective and acceptable way to identify patients who receive service at outpatient clinics. Four themes emerged, including: i) Challenges affecting current patient identification standards, ii) Healthcare workers’ views on facial recognition, iii) Perceived digital facial recognition implementation challenges, and iv) Solutions to challenges of digital facial recognition. The healthcare workers recommended ensuring the protection patients’ images privacy, providing adequate technological infrastructure in clinics, and securing stable internet access for the successful implementation of digital facial recognition.

**Conclusion:**

Our exploratory study indicates that overall, healthcare workers have a positive perception of the digital facial recognition application. However, it is crucial to acknowledge and address concerns regarding confidentiality and privacy to pave the way for the future implementation of the system.

## Introduction

Misidentification of patients is a significant problem in healthcare systems, especially in low-income countries. Misidentification can lead to medication errors, transfusion errors, testing errors, and wrong surgical procedures [1]. In the United Kingdom, there are approximately 12,000 incidents of misidentification annually, with approximately 900 causing harm [2]. Unique patient identification (UPI) is widely utilized in health systems, especially in high-income countries, as a way of preventing misidentification and medical errors [3]. This ensures the safe delivery of high-quality healthcare [3]. However, UPI remains a challenge in the healthcare systems of most low- and middle-income countries [4,5]. Patient identification is the process of correctly matching a patient to appropriately intended interventions and communicating information about the patient’s identity accurately and reliably throughout the continuum of care [6]. Patient misidentification occurs across various clinical areas, including medical imaging [7], drug administration, phlebotomy, blood transfusion, and surgical interventions [8]. Correct patient identification remains an essential aspect of safe and high-quality healthcare [9], regardless of location.

Globally, misidentification of patients is classified as a medical error and can result in various poor health outcomes [10], the most severe being death. A survey of healthcare executives in the United States, conducted as part of the 2016 National Patient Misidentification Report, revealed that 64% claimed patient misidentification errors occurred more frequently than the reported industry standard of 8–10%. The World Health Organization (WHO) recognized the need for improved accuracy in patient identification and has recommended the use of multiple patient identification mechanisms [11,12]. As a result, countries such as the United States, New Zealand, and China utilize a standard system by assigning each patient a unique patient identification number [4]. Other countries, such as Singapore, use individual resident identity cards that act as UPI. The fundamental justification for establishing a robust, unique patient identification (UPI) system lies in the critical need to enhance patient safety [4,13]. Patient misidentification is not a minor administrative error; it is a direct cause of preventable adverse events that can lead to significant patient harm, lifelong disability, or even death [13]. While advanced biometric technologies like facial recognition introduce valid and important concerns regarding data privacy and confidentiality [14,15], the ethical imperative to protect patients from such catastrophic outcomes provides a powerful motivation to explore more accurate and reliable identification systems. This study, therefore, proceeds from the premise that the profound benefits of eliminating life-threatening medical errors warrant a thorough investigation of these technologies, provided that the associated privacy risks can be understood and effectively managed.

Several low and middle-income countries (LMICs), such as Kenya, Rwanda, and Uganda, have also initiated efforts to introduce electronic health records (EHRs) [11,16]. The EHR initiatives are intended to address the problem of non-standardized patient identification by establishing unique health identifications (IDs) and enhancing the accuracy of patient records [17] Despite these efforts, the adoption of EHRs has been slow due to challenges with interoperability across systems, and it does not guarantee unique identification. Many LMICs, including Uganda, still lack a standardized approach to patient identification in both outpatient and inpatient healthcare settings. Most healthcare facilities rely on either paper-based or electronic health records, or a combination of both, to capture and manage patient identification [18,19].

Healthcare providers in Uganda actively manage patient identification using a mix of electronic and paper-based systems [19]. They use Electronic Medical Record (EMR) systems within individual facilities to assign record numbers, but these numbers are only valid within those facilities [20]. Administrators implement the Health Management Information System (HMIS), a paper-based system, to collect health data, but they struggle to achieve interoperability across facilities [21]. Researchers and policymakers advocate for a more integrated solution to address these challenges [19–21]. Digital facial recognition using biometrics is increasingly incorporated into healthcare processes, including touch-free appointment check-ins and accurate patient matching [22]. These systems often leverage artificial intelligence (AI) to analyze facial features and accurately match them to a patient’s existing medical record. Technology-based biometrics offer a promising solution to address patient identification challenges [23]. A few LMICs, including Kenya, have implemented facial recognition systems that are effective in correctly identifying patients and reducing the negative outcomes associated with misidentification [5]. However, there are limited studies that have explored the perceptions and attitudes of patients and providers on the use of mobile facial recognition in Uganda [5,24,25]. Uganda, with a population of approximately 45.9 million [26], has a ratio of doctors to patients and nurses to patients around 1:25,000 and 1:11,000, respectively [27]. These figures fall significantly short of the WHO’s ideal doctor ratio of 1:1,000 [27,28]. While the WHO does not set a single universal standard for nurse-to-patient ratios, it recommends that countries use evidence-based staffing methods to determine appropriate nurse staffing according to local needs. Uganda’s nurse-to-patient ratio remains very high, especially compared to developed healthcare systems [29,30]. This highlights the need for innovative solutions like biometrics to strengthen healthcare delivery and reduce system inefficiencies [31,32]. This study aimed to explore the perceptions and attitudes of healthcare workers towards the use of a digital facial recognition application for unique patient identification in Uganda.

## Methods

### Study setting and population

This study was carried out at Uganda Martyrs Hospital Lubaga, located in Kampala, Uganda, from June 2021 to July 2021. The participants in the study were health workers, aged 20–39, who actively participated in patient identification processes.

### Study design and sampling

A cross-sectional, explorative, qualitative pilot study was conducted on health workers selected from Lubaga Hospital in Kampala, Uganda, from June 2021 to July 2021. Eligible participants included health workers aged 18 years and older who provided informed consent. Purposive sampling was used to recruit a sample balanced by gender (5 male, 5 female), aiming to capture a range of perspectives given that professional roles and tenure can be related to gender [33]. For this exploratory pilot study, our target sample was approximately 10–12 participants, a common size for identifying initial themes in qualitative health research. We concluded data collection with a final sample of 10 participants, as we observed that the final interviews were largely reiterating the primary themes from earlier interviews, suggesting we had reached a point of sufficient information for our pilot objectives. We reported this study following the COREQ (Consolidated Criteria for Reporting Qualitative Research) 32-item checklist to ensure methodological rigor in Supplementary Table 2 in S1 File.

### Interview instrument

The study employed a semi-structured interview guide, highlighting open-ended questions, to investigate healthcare providers’ perceptions and attitudes regarding the use of mobile facial recognition (MFR) technology for patient identification within the Ugandan healthcare setting. The guide aimed to gather insights into current patient identification practices, perceived challenges, and attitudes toward digital innovations such as MFR. It explored seven questions on: professional role and involvement in patient identification or digital health policy; current methods and challenges in patient identification at the facility level; awareness and perceptions of MFR technology; alignment of MFR with national health and identification priorities; perceived benefits, risks, and barriers to adoption; feasibility of integrating MFR into existing workflows; and recommendations for implementation and scale-up. Probing questions addressed key issues such as data privacy, cost, and the appropriateness of MFR in health settings.

### Prototype application

#### Description of Artificial Intelligence Mobile Facial Recognition (AIMFAR).

**Fig 1:**
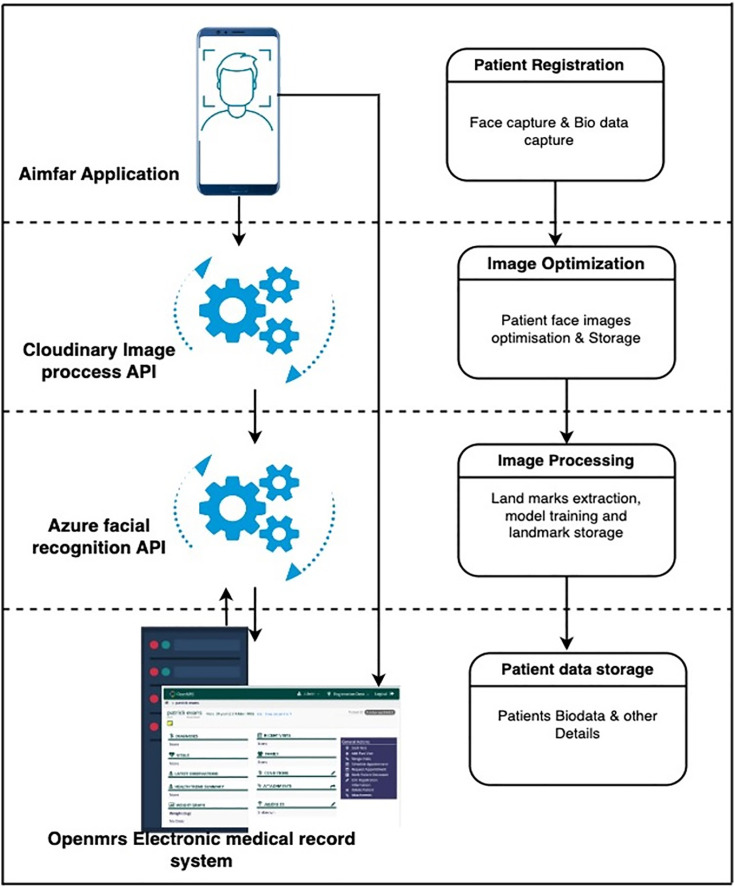
Architectural diagram of the patient identification and registration workflow. The process begins with biometric face capture in the AIMFAR Application and concludes with data storage in the OpenMRS electronic medical record system, facilitated by image processing and facial recognition APIs. **Aimfar Application (Mobile Interface):** The mobile application used for initial face and biometric data capture. **Cloudinary Image Process API & Azure Facial Recognition API (Processing Engines):** Responsible for image optimization, landmark extraction, and model training. **OpenMRS Electronic Medical Record System (Desktop Interface):** The destination system for final patient data storage and access. **Process Block (Titled Rectangle):** A major stage in the data processing workflow. **Data Storage Block (Cylinder Shape):** The final database where patient biodata and other details are stored. **Arrow:** The direction of data flow between system components.

The mobile application was developed to facilitate participant registration by capturing facial images and biodata, featuring four main functionalities. The first is a patient-facing interface, a smartphone-based facial recognition application for capturing biodata and facial images. The second is image optimization, which enhances the captured images by cropping, resizing, and adjusting for lighting. The third is image processing, involving facial landmark extraction and model training for accurate recognition. The fourth is a health worker-facing interface, providing a computer-based login to an electronic medical record system where submitted biodata and images are securely stored on a server. The application synchronizes data in real-time with the medical system when connected to the internet and supports offline internet functionality by temporarily storing data locally and automatically syncing it once internet connectivity is restored, as shown in Fig 1.

### Data collection

We conducted key informant interviews (KIIs) to assess perceptions and attitudes toward using mobile facial recognition technology for patient identification in healthcare. After obtaining written informed consent from each participant, our trained interviewer conducted the session in person. We audio-recorded each interview, which lasted approximately 45–60 minutes, for subsequent analysis. During the interviews, we used a semi-structured guide to explore topics such as current identification challenges, perspectives on the prototype application, and potential barriers and benefits to its implementation (see Supplementary Materials in S1 File for the full guide).

### Interview approach

All interviews were conducted in person at the participants’ workplaces for their convenience. Before beginning each session, we obtained written informed consent. To provide concrete context for the subsequent questions, we then delivered a brief (approximately 5–10 minute) demonstration of the AIMFAR application on a smartphone. It is important to note that the application was used as a demonstration tool for this study and was not previously deployed in the hospital’s live workflow. The demonstration included registering a fictional patient, capturing a facial image, and retrieving the record via facial scan. Following this demonstration, we proceeded with the main audio-recorded interview, which lasted between 40 and 60 minutes.

### Data management

The audio-recorded interviews of KIIs, the transcripts, master coded sheets, and all other data forms were stored under a password-protected folder on a laptop, and a copy was saved on an external hard drive as a backup. File naming and data tracks were created to access the files; these included participant identification (ID) numbers, the interviewer’s name, the type of data collection method, and the data collection date. All interviews were transcribed verbatim, ensuring participants’ confidentiality. The KII transcripts were labeled using the profession, data collection method, and participant order of interview, i.e., Nurse, KII001 for the first healthcare interview. The Principal Investigator (PI) supervised data collection and reviewed transcripts for quality and accuracy. At the outset of data collection, the PI listened to a sample of audio recordings to assess the completeness and quality of the information gathered. Then further reviewed the transcripts for accuracy, and all inconsistencies were corrected accordingly.

### Data analysis

We manually analyzed data from the KIIs using thematic analysis, drawing on the principles outlined by Braun and Clarke [34]. The iterative process was both deductive, using interview questions to guide initial coding, and inductive, allowing themes to emerge directly from the data. A qualitative data analysis expert reviewed the coding process to ensure rigor. Our analysis was conducted primarily at a semantic level to summarize the explicit content of the participants’ responses. This descriptive approach was deliberately chosen to align with the exploratory goal of this pilot study, which was to map the key perceptions and attitudes articulated by healthcare workers. To provide an overview of theme prevalence, we tabulated the frequency of each code, defined as the number of unique participants who discussed the concept (see Supplementary Table 1 in S1 File). However, consistent with our qualitative methodology, the analysis focused on interpreting the meaning of themes across the entire dataset. We did not perform a quantitative comparison of code frequencies between participant subgroups, as this would not be methodologically sound with our small sample size. Finally, the prominent emerging themes and subthemes were generated and finalized.

### Ethical considerations

The study was approved by the Higher Degrees Research and Ethics Committee (HDREC), School of Public Health, and Uganda Martyrs Hospital Lubaga management. Consent was also sought from all the participants enrolled for key informant interviews (KIIs), and participants signed consent forms before participating in the study. Participants were reimbursed for their time spent on the day of the interview.

## Results

### Characteristics of participants

A total of 10 interviews were conducted among doctors and nurses with an equal number of males and females. The majority were aged 20–29 and had worked at the hospital for more than one year, and all participants had attained tertiary education, as summarized in Table 1.

**Table 1:** Characteristics of participants.

Participant Characteristics	KII Participants n (%)
**Gender**	
Male	5 (50)
Female	5 (50)
**Age**	
20-29	6 (60)
30-34	2 (20)
35-39	2 (20)
**Education level**	
No formal education	–
Primary	–
Secondary	–
Tertiary	10 (100)
**Job description**	
Nurses	5 (50)
Doctors	5 (50)
**Duration in profession**	
1–2 years	2 (20)
3–5 years	7 (70)
Above 5 years	1 (10)

### Summary of key findings

Healthcare workers identified several challenges that impact current patient identification systems. They struggled to retrieve paper-based records, faced confusion caused by similar or misspelled patient names, dealing with changes in standard identifiers like phone numbers or addresses, and encountered failures in electronic systems due to internet or power outages. They emphasized poor data management, particularly for pediatric patients. Participants mentioned mobile facial recognition as a promising solution, highlighting its ease of use, improved accuracy, reduced delays in patient care, and enhanced support for patient tracking. They also expressed concerns about privacy, confidentiality, technology infrastructure, hardware availability, and implementation costs. To address these issues, they proposed strengthening internet access, collaborating with telecom providers, and raising public awareness to tackle ethical concerns.

### Perceptions and attitudes of healthcare workers towards the use of mobile facial recognition applications in health settings

#### Challenges affecting current patient identification standards.

Healthcare workers identified the following challenges affecting the identification of patients.

#### Retrieving patient records from paper medical registers.

Almost all participants mentioned the issue of revisiting the register every time a patient comes to the health Centre. Health providers explained that retrieving patients’ biodata during return visits is challenging and time-consuming, particularly when patients lack previous records.

*“The challenge we face is when patients come back for review, we always have to revisit the register to retrieve their information and cross-check using biodata.”* (Nurse, KII001)

*“Sometimes, patients return without their receipts, and we have to use their names for identification. At times, patients might share names with someone else. As a result, we may end up with the wrong person, who then opens a new PIN number because we cannot locate their old one.”* (Nurse, KI008)

#### Identification of correct patient names.

Half of the participants explained that during identification, they encounter challenges with patients who have the same or misspelled names or names that are otherwise mistaken.

*“We have a system where each patient is attached to an identification number, although, at times, we encounter people who share names. We have tried to solve that issue by using different methods, like using telephone numbers. Moreover, sometimes you might have people with misspelled names*.” (Nurse, KII05)

#### Change of standard used identifiers.

Some participants highlighted challenges caused by patients changing their identifiers. Participants noted that patients often change their cell phone numbers, village, or location.

*“If I last visited the hospital, let’s say two years ago, and I come back and I happen to have changed my phone number, I am not easily identified as a person who has ever visited the hospital because I am presenting a new phone number.”* (Doctor, KII04)

#### Failure of the electronic identification system.

Some participants highlighted challenges with the electronic system, which fails to function properly during internet outages. This prevents the system from loading patient records or generating ID numbers.

*“The challenge that we face is when there is a system failure. In that case, everything is faulty. Even if you see a patient, you can’t give an identification number because everything works through the system. And the system is internet-based, so if the internet is down or if the application is down, then the whole system will be down.”* (Doctor, KII04)

#### Poor data management.

In the current identification system, poor data management is a challenge, especially when child patients are registered under their parents instead of having individual medical records.

*“Especially for children, their records are always linked to their parents. Knowing the next of kin is good, but children are not registered as individuals. It would be better if this child had his/her medical record.”* (Doctor, KII04)

### Healthcare workers’ views on facial recognition

#### Ease of use in the patient identification process.

Most of the participants believed that facial recognition would be easy to use in the identification process. They noted it would require minimal steps to display data immediately, reducing delays in care. A benefit of the ease of use reported was reduced delays for patients to receive care. Some participants expressed the following views.

*“The use of digital facial recognition seems to be faster as compared to electronic/hardcopy existing systems used, since there is a lot of writing, typing, and continuous meetings with the patient asking for the same details.”* (Nurse, KII01)

*“It’s beneficial because it will simplify identification in hospitals. When a patient arrives, instead of searching for details, staff can just scan a face, and their data is immediately displayed; this simplifies work in the hospital, allows people to leave quickly, and reduces delays.”* (Doctor, KII07)

#### Improved patient care.

Most participants believed facial recognition would improve patient care through easy and accurate identification. They noted benefits like accurate treatment, fewer medical errors, and reduced triage delays.

*“The use of technology would help the management team to give the right treatment to the right patient.”* (Doctor, KII03)

*“It reduces making mistakes when dispensing drugs to the wrong patient or interchanging their drugs.”* (Doctor, KII02)

#### Improved patient identification.

More than half of the participants mentioned that the facial recognition system will improve patient identification during the identification process. They highlighted that it would remove the need to revisit patient records, making the identification process simpler. Participants also noted that facial features remain constant, allowing quick and reliable data access via scanning.

*“It’s not something bad because it’s going to ease work in the hospitals. Because when the patient comes, instead of looking for the details, they just scan immediately, and my data is out, it will ease work in the hospital and people will be able to leave quickly, reducing delay.”* (Doctor, KII06).

#### Support for use in health facilities.

Almost all the participants were of the view that mobile facial recognition identification can be used in healthcare facilities. Participants believed it would be appreciated by staff and patients, enhance efficiency, and simplify tracking of unpaid bills. Some participants suggested it might be more practical to implement in private hospitals due to their better resources and infrastructure. This is explained by the following comments from two KI interviewees.

*“Yes, because of the modernized world, everything is changing. There could be a chance, maybe in the future, because at first, we used to record in books only, but today we have an electronic system. The use of mobile facial recognition is better.”* (Nurse, KII01)

*“Yes, everything is possible, but you have to first look at the cost of using the system, user-friendly, and whether it is a tedious process because in a health facility, we don’t want things that give us extra work.”* (Doctor, KII03)

### Perceived digital facial recognition implementation challenges

#### Privacy and confidentiality concerns.

Some participants raised the challenge of ethics in the implementation of digital facial recognition in healthcare facilities. They noted that some patients might resist having their photos taken. Additionally, some participants expressed concerns about confidentiality and privacy. Participants expressed concerns that ethical considerations and issues related to confidentiality and privacy could affect patient acceptance of the system.

“*And then my other concern is the confidentiality of this information. As medical people, we really want confidentiality and privacy of this information to be taken seriously.”* (Doctor, KII08)

*“Willingness and cooperation of patients, patients might have issues taking their pictures, so there is a need to explain to them about the importance, which may take a lot of time.”* (Doctor, KII02)

#### Technology support infrastructure.

One of the challenges likely to be incurred in the implementation of digital facial recognition is the unavailability of the required technology infrastructure. Participants described several related challenges such as unreliable internet connection, inadequate internet network coverage, system, or equipment breakdown, and instability of electricity. The views were expressed by one participant as follows.

*“I don’t know if data is needed; if needed, network issues can arise in case the health center is deep in the village where network coverage is a problem.”* (Nurse, KII01)

#### Adequate and durable hardware.

The other challenge identified was hardware issues, and this was explained as the durability of devices, several smartphones or tablets may be needed, and the cost of server infrastructure.

*“A number of smartphones or tablets might be needed due to different stations in the clinic or hospital, hence being costly.”* (Doctor, KII02)

#### Cost to sustain the digital system.

The implementation of digital facial recognition may be cost-intensive because of the upfront investment that is needed in the facilitation of staff training, the cost of internet, and the cost of hardware, as described by half of the participants who answered this question.

*“There is a need for facilitation of staff training, and smartphones are needed because not every phone can allow the application to be installed.”* (Nurse, KII05)

### Solutions to the challenges of digital facial recognition

#### Infrastructure challenges.

Some participants proposed solutions such as ensuring stable internet, hiring internet service providers, and connecting health facilities to the national power grid.

*“The government must find means of putting in place a stable internet in all health centers.”* (Nurse, KII01)

#### Cost of implementation.

One participant suggested partnering with telecom companies to subsidize or provide free data through corporate social responsibility.

*“Talking to telecom companies, maybe corporate social responsibility contributing data or subsidizing the data bundles.”* (Doctor, KII03)

### Ethics

On the issue of ethics, one suggested raising public awareness of digital facial recognition through radio and TV announcements.

*“By creating awareness in the public, depending on different villages, or also an announcement on radio and television.”* (Doctor, KII06)

## Discussion

This study explored the perceptions and attitudes of healthcare workers in Kampala, Uganda, towards the use of digital facial recognition technology for patient identification. Overall, the findings revealed a generally positive outlook among healthcare workers regarding the potential benefits of facial recognition systems. However, some key challenges were perceived to be related to privacy, infrastructure, and costs. These findings provide important insights into the feasibility and acceptability of implementing such technology in low-resource healthcare settings [35,36].

Participants expressed confidence about the potential for facial recognition technology to simplify patient identification processes, reduce delays, and improve care delivery. By eliminating the reliance on paper-based registers and reducing errors in patient identification, the technology was seen to enhance efficiency and minimize medical errors. This aligns with findings from other studies that have demonstrated the effectiveness of facial recognition systems in healthcare to improve accuracy and patient safety [13,37–39]. Furthermore, healthcare workers noted that the often-consistent nature of facial features in adults offers a reliable solution to challenges such as duplicate records or changes in personal identifiers. These benefits are consistent with prior research on facial recognition for patient identification in healthcare [24,40].

In contrast, some participants raised concerns about privacy and confidentiality arising from the storage and use of patients’ images, which they felt could undermine acceptance of the technology. These findings are consistent with other studies on biometrics, which also document that secure and ethical data use is critical for acceptance [41–43]. Importantly, these concerns are not merely theoretical but are directly addressed by Uganda’s national legal framework, making their resolution a legal prerequisite for implementation. The Data Protection and Privacy Act, 2019 [44], provides the primary governance, classifying health data as sensitive personal data that requires explicit patient consent for collection. The Act legally mandates robust data security measures and establishes significant penalties, including fines and imprisonment, for misuse. This is reinforced by the Ministry of Health’s Patients’ Charter [45], which guarantees the right to confidentiality. Therefore, any successful implementation must involve clear communication with patients about their legal rights under these policies, alongside comprehensive training for staff to ensure strict compliance and minimize ethical risks [46,47].

Healthcare workers noted that unreliable internet connectivity, inadequate power supply, and the need for durable hardware could impede the implementation of facial recognition systems. These challenges are consistent with other findings from LMICs, where resource limitations often hinder the adoption of digital health technologies [5,8] Innovative approaches, such as partnering with telecommunications companies to provide subsidized internet or leveraging corporate social responsibility initiatives, could help mitigate some of these barriers. Additionally, piloting the technology in private hospitals or urban centers with more stable infrastructure could further strengthen the proof of concept. Finally, it is important to consider these findings within the context of the used methodology. We employed an exploratory qualitative design as this was a pilot study intended to generate initial insights rather than generalizable statistics. The focus on healthcare workers was a deliberate first step to gauge the perspectives of the technology’s primary users and implementers. Similarly, the use of a brief technology demonstration, rather than a long-term deployment, was a practical approach to ensure all participants had a consistent baseline understanding from which to form their initial perceptions. This methodological framework is essential for framing our results as a foundational exploration of stakeholder receptivity, which naturally leads to the specific strengths and limitations of the study.

### Strengths and limitations

A key strength of this study is that it provides preliminary insights into healthcare workers’ perspectives on the use of facial recognition technology for patient identification in a low-resource setting. As one of the first studies to explore this topic in Uganda, it can catalyze further research.

However, the primary limitation of this study is its exclusive focus on healthcare workers. While this was intentional for a pilot study centered on implementation feasibility from the provider’s viewpoint, the absence of patient perspectives provides an incomplete picture. Patients are the ultimate end-users of this technology, and their views on privacy, trust, consent, and usability are important. Therefore, the findings reported here should not be interpreted as a comprehensive assessment of the technology’s overall acceptability, but rather as a foundational analysis of provider-side readiness. The findings should be considered in light of three further limitations. First, the small sample, drawn from a single facility, limits the generalizability of our results and precludes a sub-analysis of how perceptions might differ by professional role or experience. Second, we assessed reactions to a brief technology demonstration, which may not reflect the practical challenges or long-term attitudes that would emerge during real-world implementation. Third, participants’ varying familiarity with existing paper and electronic systems may have influenced their perceptions of this novel technology [19]. Nevertheless, the study still provides valuable considerations for the implementation of facial recognition technology within health facilities in low-resource settings.

## Conclusion

Overall, healthcare workers in this study expressed optimism about the potential benefits of digital facial recognition technology for patient identification, while also highlighting important concerns around privacy and infrastructure challenges. Addressing these issues will be crucial if such technologies are to be successfully implemented in the context of the Ugandan healthcare system. Further research involving larger and more diverse samples of healthcare providers and patients is needed to better understand the complex factors influencing the acceptability and feasibility of facial recognition systems in low-resource healthcare settings.

## Supporting information

S1 File Supporting materials include the consent form, key informant interview guide, summary of qualitative themes, and the COREQ checklist. (PDF)
