# Supplementary material for: Perceptions and attitudes of healthcare workers towards the use of digital facial recognition application in a health setting in Uganda: An exploratory pilot study
Source: PLoS One. 2025 Nov 25;20(11):e0337691. doi: 10.1371/journal.pone.0337691 (PMC12646429; doi:10.1371/journal.pone.0337691)
Supplement: S1 File — (PDF) [file pone.0337691.s001.pdf]

A Mobile Facial Recognition Application for Unique Identification and Matching of Patients in Healthcare and Community Settings in Uganda. Health Providers Key Informant Interviews Consent

Principal Investigator: XXXXXX, student of health informatics at Makerere University School of Public Health. I am conducting a research in Partial Fulfillment of the Requirements for the Award of Degree of Master of Health Informatics, by the School of Public Health Makerere University.

Background

Unique Patient Identification (UPI) continues to be a challenge in the health sector in Uganda. Correct patient identification remains an important aspect of safe and high-quality health care. Failure to correctly identify patients through the health care sector results in direct patient harm, medication errors, diagnosis errors, procedures on wrong persons, discharge of infants to wrong families, stress and anxiety for patients, and time pressure for health staff.

Purpose of the Study

I am conducting this research study to assess the performance of a new approach of using smartphones and facial recognition algorithms to carry unique patient identification and matching of their medical records to improve patient health care. I want to know if the facial recognition method will be more effective in uniquely Makerere University, School of public Health, Master of Health Informatics

Hello, my name is **XXXXX.** I am master's identifying patients. The information we obtain could be used to design a system that could be used by the National identification program to improve the way patients are identified while they are seeking treatment. Your participation in this research study is entirely voluntary.

Before trying this method, we want healthcare providers to give us their feedback on this idea. To gather as many ideas as possible, I am holding individual interviews from key informants. You

have been identified as a key informant because of your leadership position in the healthcare. I invite you to voluntarily participate in this interview because I believe you will contribute valuable information that we need to design an effective mobile-phone based facial recognition system to support patient identification.

Procedures

If you agree to participate in this Study, you will be asked to:

1. Be interviewed in-depth about your opinions and perceptions about the new approach for mobile phone-based facial recognition system. The session will take about 1 hour.

2. Listen to a brief presentation describing how mobile facial recognition works.

3. Share your personal experiences about how the current usual patient identification in health facilities.

4. Share your opinions about potential problems and benefits that could come from using this new mobile facial recognition for identifying patients in seeking healthcare.

5. Be audio recorded throughout the in-depth interview.

6. Complete a brief survey lasting about 5 minutes after the interview. The survey will ask general questions about yourself, your experience with using cell phones and acceptability of mobile facial recognition as a method

Audio Recording Release Consent

As part of this project, an audio recording will be made of this interview. This is completely voluntary. In any use of the audio information, your name will not be identified. If you do not want to be recorded, then you should not participate in the interview. The audio-recordings will be retained for the length of time until completion of data analyses and publication. We estimate that this will be about 2 years after the end of the Study. The following will be done:

1. The audio information will be studied by the principal Investigator to answer the research questions

2. The audio information will be transcribed and used for scientific publications.
3. The excerpts or quotes from the transcripts could be reviewed in public scientific and nonscientific presentations as necessary.

After the interview, the audio information will be transcribed into an electronic document but only unique assigned ID and no names will be included. Only designated individuals will have access to the audio records for purpose of review in relation to the research questions.

Risks/Discomforts

Taking part in this Study is anticipated to have very minimal risk and discomfort to you. To safeguard against loss of confidentiality, all interview transcripts will use unique study ID numbers to ensure privacy and confidentiality. You may also find it hard to stay for a 1-hour session.

Benefits

There is a small but immediate direct benefit from participating in this Study in that you will be reimbursed for your time and you will receive a snack and drink during the interview. Long-term, we expect that the Study may accrue indirect benefits to the health providers, the health system and the patient. The results obtained may help the researchers design an alternative method of delivering greater support in patient identification reducing the workload for providers and convenience for the patients. Mobile facial recognition has also been shown to allow flexibility in the use of the system. For example, a health provider can use the system both in a health facility and in outreaches to uniquely identify patient and enroll the patient.

Costs and Reimbursement

There will be transportation cost associated if the participant incurs cost to the interview venue of 20,000 Ugs. But if the interview is taken at the work at place of work, a refreshment will be provided. The only cost will be that of your time you are going to spend participating in the interview. This is to reimburse you for time for being in this Study.

Privacy and Confidentiality Statement

The research records will also be kept private to the extent allowed by the Uganda law. We cannot guarantee complete privacy but we will take steps to protect your private information.

To protect your privacy, we will give you a unique study ID number so your name will not be on the survey form. We ask you for your name and contact information for purposes of reaching out to you in case we would like to inform you about new developments from the Study or about the results of the interview. Electronic audio files will be kept at external hard disk for purposes of checking accuracy of the data during the transcription phase, analysis, interpretation and scientific writing. The information will be used or shared after the identifiers have been removed, for example with other researchers and/or for future studies without additional consent.

Audio files from the interview will be transferred to password protected computer with the session date on them. Only study principal investigator will listen to files. Electronic files will be reviewed only privately to allowed individuals. We will ask that participants do not use names during the interview to protect their privacy, and we will replace any names with codes when the audio files are transcribed. All personal identifying information will be removed. The typed transcripts will be kept in a locked file drawer separate from the audio files. The transcripts and audio information will be kept as source documents for a period of at least 3 years after the end of Study as required by the principle Investigator.

Termination from this Study Without your Consent

Because of the scientific nature of the Study, the researchers may also end your involvement in this Study at any time. You may be removed from the Study and referred to other resources if:

- the researcher team decides that your involvement in the interview would not be appropriate.
- you disrupt the interview in a way that the researcher considers inappropriately.

Voluntary Participation and Withdrawal Statement

You are taking part in this research study as a volunteer. If you decide to take part, you are free to take back your consent and stop being part of the Study at any time. If you decide to stop or withdraw from the Study or the Investigator terminates your participation, the information/data collected from or about you up to the point of your withdrawal will be kept as part of the Study and may continue to be analyzed. If you stop, there is no penalty to you. Taking part in this Study is up to you.

Question related to the Study

If you have questions about the Study you may call Mr. Patrick Kaggwa at 0787655016/ 0701808066.

Signature Page

By signing this consent form, I am not giving up any of my legal rights. I have received a copy of this consent form to keep. My signature below shows that I am at least 18 years old and that I voluntarily chose to participate in this Study.

If you agree to participate in the interview and to comply with all procedures, please sign your name below

_____________________________ _________________________ ___/___/___

Name of Researcher Signature Date (mm/dd/yy)

_____________________________ _________________________ __/___/_____

Name of Participant Signature or thumb print (if unable to write) Date (mm/dd/yy)

Please keep one copy and return the signed copy to the researcher.

KEY INFORMANT GUIDE FOR TB HEALTH WORKERS

Introduction

I appreciate your help with the consent form. A few notes about our session today. This session will be audio recorded, but your name will not appear in any report or summary of the session. All of your comments are very important and we would like to give each person the opportunity to talk about their experiences or express their views. It is also important to remember that what is talked about here is confidential and that we respect everyone's privacy and keep our conversation in this room.

Ok, now let's get started…

People who work in health care know it is important to be able to uniquely identify patients for proper patient care. Properly identifying patients has shown improved patient care and safety, but most hospital of the hospitals are not fully using electronic medical records systems; some are still using hand copy or both. We want to understand better whether we can use cell phone technology to uniquely identify patients visiting the health facility.

I will now explain how mobile facial recognition is done and how it works**. (EXPLAIN MOBILE FACIAL RECOGNITION PROCESSWITH A DIAGRAM BRIEFLY TO PARTICPANTS).**

MOBILE FACIAL RECOGNITION: Are they appropriate? What does the policy say?

Qn: Briefly explain your (professional) role and the kind of work you do?

Qn: What role do you play in developing or implementing policies related to digital technologies in Kampala and the whole country?

Qn: What in your view, are the priorities for investing resources to putting unique identification in Uganda?

Qn: In your view, what are some of the challenges the hospital faces in identification of **Probe:** Specifically, how is the hospital is carrying out patient identification.

Qn: What do you know about the use of technology in patient identification? **Probe:** What about what about mobile facial recognition?

Qn: What are your personal views about the use of mobile facial recognition?

Qn: How does mobile facial recognition as a patient identification strategy fit in the current priorities of the Uganda National identification program?

**Qn:** How do you see mobile facial recognition in relation to the current standard used in patient identification? **Probe:** to compare mobile facial recognition with current standards used in patient identification.<br>**Qn:** Do you think there is a place for using **mobile facial recognition technology** for patient identification in health facilities in Uganda? Why or why not?<br>**Qn:** Do you think mobile facial recognition services should be made available to health workers in Uganda? Which people should use mobile facial recognition? **Probe:** In what situations do you think mobile facial recognition would not be good to use?<br>**Qn:** What do you see as the major challenge(s) to making mobile facial recognition more widely used by health facilities in Uganda?<br>**Qn:** How can these challenges be overcome if at all?<br>**Qn:** Talk about the likely cost of mobile facial recognition use in Uganda.<br>**Qn:** How do the patient identification guidelines in Uganda may support the use of mobile facial recognition technology among patients?<br>**Qn:** How could mobile facial recognition technology be incorporated into the existing patient identification procedures?<br>**Qn:** Is there anything else I should know to better understand identification policies and how they may affect the use of mobile facial recognition technology?
**Final Thoughts - End of the Session**
**Qn:** What other ideas do you think could help health facilites use the smart phone for uniquely identifying patients? Perhaps something that we have not talked about yet, that may improve our ability to use this technology for patient unique identification?
**End of Session**
Conclusion: This concludes our interview. I very much appreciate your participation today. What we have learned from you is very important in order to know how to best implement the mobile facial recognition Pilot program in our health facilities. **Once again, thanks.**

Table 1: Summary of themes and codes

Theme	Codes	Frequency
**Challenges Affecting Current Patient Identification Standards**	Retrieving patient records from paper medical registers	9
	Identification of correct patient names	5
	Change of standard used identifiers	4
	Failure of electronic identification system	6
	Poor data management	7
**Healthcare Workers' Views on Facial Recognition**	Ease of use in the patient identification process	8
	Improved patient care	8
	Improved patient identification	7
	Support of use in health facilities	9
**Perceived Digital Facial Recognition Implementation Challenges**	Privacy and confidentiality concerns	4
	Technology support infrastructure	2
	Adequate and durable hardware	3
	Cost to sustain the digital system	5
**Solutions to Challenges of Digital Facial Recognition**	Infrastructure challenges	2
	Cost of implementation	1
	Ethics	1

Table 2: COREQ (Consolidated criteria for Reporting Qualitative research) Checklist			
**Topic**	**Item No.**	**Guide Questions / Description**	**Reported on Page No.**
**Domain 1: Research team and reflexivity**			
**Personal characteristics**			
Interviewer/facilitator	1	Which author/s conducted the interview or focus group?	Page 1, 31
Credentials	2	What were the researcher's credentials? e.g., PhD, MD	Page 1, 31
Occupation	3	What was their occupation at the time of the study?	Page 1, 31
Gender	4	Was the researcher male or female?	Page 1, 31
Experience and training	5	What experience or training did the researcher have?	Pages 9–10, Methods, Data collection
**Relationship with participants**			
Relationship established	6	Was a relationship established prior to study commencement?	Pages 9–10, Consent/Methods
Participant knowledge of the interviewer	7	What did the participants know about the researcher?	NA
Interviewer characteristics	8	What characteristics were reported about the interviewer/facilitator?	NA
**Domain 2: Study design**			
**Theoretical framework**			
Methodological orientation and theory	9	What methodological orientation was stated to underpin the study?	Page 10, Methods, Data Analysis
**Participant selection**			
Sampling	10	How were participants selected?	Page 7, Methods, Study Design/Sampling
Method of approach	11	How were participants approached?	Pages 8–9, Methods, Data Collection
Sample size	12	How many participants were in the study?	Pages 7, 11, Results, Table 1
Non-participation	13	How many people refused to participate or dropped out?	NA
**Setting**			
Setting of data collection	14	Where was the data collected?	Pages 7, Methods, Study Setting
Presence of nonparticipants	15	Was anyone else present besides the participants and researchers?	NA
Description of sample	16	What are the important characteristics of the sample?	Page 11, Results, Table 1
**Data collection**			

Interview guide	17	Were questions, prompts, guides provided by the authors? Was it pilot tested?	Pages 7–8, Methods, Data Collection
Repeat interviews	18	Were repeat interviews carried out?	NA
Audio/visual recording	19	Did the research use audio or visual recording to collect the data?	Page 9, Methods, Data Collection
Field notes	20	Were field notes made during and/or after the interview or focus group?	NA
Duration	21	What was the duration of the interviews or focus group?	Pages 9, Methods, Data Collection
Data saturation	22	Was data saturation discussed?	Page 7, Methods, Study Design/Sampling
Transcripts returned	23	Were transcripts returned to participants for comment and/or correction?	NA
**Domain 3: Analysis and findings**			
**Data analysis**			
Number of data coders	24	How many data coders coded the data?	Page 10, Methods, Data Analysis
Description of the coding tree	25	Did authors provide a description of the coding tree?	Supplementary, summary of themes Table1.
Derivation of themes	26	Were themes identified in advance or derived from the data?	Page 10, Methods, Data Analysis
Software	27	What software, if applicable, was used to manage the data?	Page 10, Methods, Data Analysis
Participant checking	28	Did participants provide feedback on the findings?	NA
**Reporting**			
Quotations presented	29	Were participant quotations presented to illustrate the themes/findings?	Pages 11–19, Results, Discussion
Data and findings consistent	30	Was there consistency between the data presented and the findings?	Pages 11–22, Results, Discussion
Clarity of major themes	31	Were major themes clearly presented in the findings?	Pages 12–19, Results, Discussion
Clarity of minor themes	32	Is there a description of diverse cases or discussion of minor themes?	Pages 13–19, Results, Discussion
